# *Blautia* genus enrichment following a multidomain intervention and its association with gut microbiota and psychosocial parameters in cognitive frail older adults

**DOI:** 10.1002/alz70860_106306

**Published:** 2025-12-23

**Authors:** Hazwanie Iliana Hairul Hisham, Siong Meng Lim, Kalavathy Ramasamy, Abu Bakar Abdul Majeed, Suzana Shahar

**Affiliations:** ^1^ Collaborative Drug Discovery Research (CDDR) Group, Faculty of Pharmacy, Universiti Teknologi MARA (UiTM), Puncak Alam, Selangor, Malaysia; ^2^ Brain Degeneration and Therapeutics Group, Faculty of Pharmacy, Universiti Teknologi MARA (UiTM), Puncak Alam, Selangor, Malaysia; ^3^ Centre for Healthy Aging and Wellness, Faculty of Health Sciences, Universiti Kebangsaan Malaysia, Kuala Lumpur, Malaysia

## Abstract

**Background:**

Cognitive frailty (CF), a condition characterised by the co‐occurrence of physical frailty and cognitive impairment, has been linked to dysregulated gut microbiota (i.e., dysbiosis) and increased intestinal permeability. Emerging evidence suggests that the gut microbiome plays a crucial role in maintenance of gut‐brain barrier integrity, suppression of neuroinflammation, regulation of neurotransmitter production, and ultimately promotion of psychological health. This study aimed at examining the effects of a 6‐month multidomain intervention (i.e., physical exercise, cognitive training, psychosocial training and nutritional guidance) on gut microbiota composition.

**Method:**

A total of 27 CF participants (12 control; 15 intervention) were recruited and stool samples were collected for 16S rRNA sequencing. The stool samples were homogenised, and DNA was extracted, followed by PCR amplification, DNA library preparation, and sequencing. Statistical analysis was performed using GraphPad Prism. Normality was assessed using the Shapiro‐Wilk Test, and differences between groups were analysed using the Ordinary One‐way ANOVA Test. Correlations between genus abundance and psychosocial parameters were evaluated using Pearson's correlation analysis.

**Result:**

Microbiota profiling revealed a significant increase of the *Blautia* genus in the intervention group (IV6, intervention after 6 months) when compared to the control group (C6, control after 6 months) (*p* = 0.0132), suggesting a positive shift in gut microbial balance following lifestyle modifications. The *Blautia* genus abundance was negatively correlated with depression (*r* = ‐0.4120, *p* = 0.0022) and loneliness scores (*r* = ‐0.3328, *p* = 0.0184), indicating its potential protective role against psychosocial factors. On the contrary, the *Prevotella* genera exhibited a positive correlation with depression scores (*r* = 0.2820, *p* = 0.04), reinforcing its association with neuroinflammation which disrupts neurotransmitter balance.

**Conclusion:**

The present results highlight the positive impact of lifestyle interventions on gut microbial composition and their possible implications for psychosocial health in older adults with CF. The *Blautia* genus enrichment may serve as a biomarker or therapeutic target for improving psychosocial well‐being in CF individuals. This warrants further investigation of the mechanistic pathways linking gut microbiota modifications with psychosocial outcomes.

**Funding:**

This study is part of the Transforming Cognitive Frailty to Later‐Life Self‐sufficiency (AGELESS) project funded by the Ministry of Higher Education, Malaysia under the Long‐Term Research Grant Scheme (LRGS/1/2019/UM/01/1/3).

